# Notes on Dyspareunia

**Published:** 1909-09-25

**Authors:** 


### Gynæcology.

### NOTES ON DYSPAREUNIA.

Dyspareunia leads not only to great physical pain in many instances, but also to great mental suffering, often ending in serious neurasthenia. Its causes are not always apparent, and require very careful examinations and a thorough scrutiny of the history of the case for their diagnosis and differentiation. The cases naturally fall under two headings, according to the presence or absence of a definite local lesion, and the prognosis in any particular case often depends upon this. It is much easier to cure those patients who have a definite painful lesion than those in whom no lesion can be demonstrated; in fact, the latter may be incurable. The former class of case is nearly always dependent upon an acquired lesion. Where no lesion can be found the history usually is that coitus has been painful from the first. Further, the pain is commonly described as getting worse as time goes on in the cases which have a lesion, whereas it remains always about the same in those without lesions. To make a diagnosis in a given case a complete examination of the genital organs must be made, commencing with abdominal palpation, going on to inspection of the external genitals, and ending with a bimanual examination of the uterus and adnexa, and a visual examination of the vagina and cervix uteri with the speculum. Further, it must be remembered that the lesion may be in the anus or rectum, and that very occasionally the urethra and bladder are at fault. Therefore it follows that these organs must be examined in any otherwise obscure case.

The commonest cause is undoubtedly inflammation of the carunculæ myrtiformes combined with a chronic vulvitis. This in many instances is gonorrhœal in origin, but although it is of interest to know this, it does not modify the prognosis or treatment to any appreciable extent. It is sufficient to realise that the lesion is one due to infection of the hymeneal remains and surrounding vulval tissues. The carunculæ look a brilliant red, and may show small ulcerated areas, whilst the surrounding tissues are infected and bathed with semi-purulent discharge. The orifices of Bartholin's glands are often swollen and prominent, a condition which is almost always present in gonorrhœal cases. The gland itself may be swollen and painful, and cystic dilatation of the duct and parts of the gland may be met with. The whole picture is one of a local chronic infection. The pain in such cases is limited to the vaginal entrance, and is most acute when any attempt at examination is made. As a rule, this represents the whole of the lesion, and no tenderness of the tubes or ovaries is found—the lesion is strictly local.

The next common cause is much more deep-seated, and lies in tender prolapsed ovaries, very often in connection with a retroflexed and retroverted uterus. Two kinds of cases present themselves, those in which the displaced uterus and ovaries are movable, replaceable, and without adhesions or inflammation, and those in which a real salpingo-oophoritis is present, with fixation of the uterus, tubes, and ovaries to the surrounding parts. The former cases are almost always set up by a previous labour or abortion (the only common causes of retroflexion and retroversion), with subsequent interference with the blood supply of the ovaries, followed by congestion and œdema and elongation of the ovarian ligament. The latter are usually the result of an infection of the tubes *via* the uterine mucous membrane, the organisms being either the gonococcus unconnected with pregnancy, or other organisms usually spreading after a septic puerperium. The cases with adhesions are the worst of all, the inflamed ovaries and tubes, bound down close to the posterior vaginal fornix, being acutely tender and intolerant of the slightest touch.

Other causes of dyspareunia occasionally met with include smallness of the vaginal orifice, urethral caruncle, fissure of the anus, thrombosed and ulcerated hæmorrhoids, urethritis, and cystitis.

When the vaginal orifice is very small and the hymen has sharp, tough edges, coitus may be impossible, and repeated attempts always give rise to pain. In these cases the mucous membrane may not be tender to a gentle touch, and, as a rule, there is no inflammation. Urethral caruncles are evident on inspecting the vulva, and may be most acutely tender to the touch. Fissure of the anus is easily discovered by examining the anus visually whilst the patient is asked to bear down. It may give rise to agonising pain on defæcation or on any disturbance of the pelvic floor such as would occur on an attempt at coitus. Thrombosed and ulcerated piles are nearly always external or can easily be made to protrude. These, of course, do not as a rule last very long, as they tend to cure themselves, but whilst they last may be acutely tender and cause dyspareunia. Urethritis and cystitis cause dyspareunia because the tender urethral and bladder walls evoke painful sensations upon movement of any of the pelvic organs.

Whilst all these lesions cause pain it must not be forgotten that they may also cause spasm of the pelvic floor muscles leading to closure of the vagina, and the condition known as vaginismus may depend upon any of these painful local lesions. Vaginismus, however, may occur without any demonstrable local lesion, in which case the spasm prevents coitus and attempts may become painful.

Treatment is fortunately eminently successful when local lesions can be demonstrated. Inflamed carunculæ are best treated by complete excision of the hymeneal ring, with careful suture, so as to obtain primary union. Painful prolapsed ovaries usually call for operative treatment, for pessaries cannot as a rule be worn, and often fail to correct the displacements. When a salpingo-oophoritis is present operations must be done, and other local lesions call for their appropriate treatment. Where no local lesion exists and the vaginal entrance is wide dyspareunia seems practically incurable.

